# Comparative Studies on Patient Safety Culture to Strengthen Health Systems Among Southeast Asian Countries

**DOI:** 10.3389/fpubh.2020.600216

**Published:** 2021-01-12

**Authors:** Sunjoo Kang, Trang Thi Thuy Ho, Nam-Ju Lee

**Affiliations:** ^1^College of Nursing, Seoul National University, Seoul, South Korea; ^2^Graduate School of Public Health, Yonsei University, Seoul, South Korea; ^3^Department of Nursing, Hue University of Medicine and Pharmacy, Hue University, Hue, Vietnam; ^4^The Research Institute of Nursing Science, Seoul National University, Seoul, South Korea

**Keywords:** patient safety, outcome, health system, Southeast Asia, safety culture

## Abstract

Patient safety is an important issue in health systems worldwide. A systematic review of previous studies on patient safety culture in Southeast Asian countries is necessary for South Korea's partnership with these countries, especially given South Korea's assistance in strengthening the health systems of these developing countries. Studies on patient safety culture in Southeast Asian countries, published in English and Thai languages, were retrieved from computerized databases using keywords through a manual search. Data extraction, quality assessment, and analyses were performed using several tools. The review included 21 studies conducted in Indonesia (*n* = 8), Thailand (*n* = 5), Malaysia (*n* = 3), Vietnam (*n* = 2), Singapore (*n* = 1), and the Philippines (*n* = 1). They were analyzed and categorized into 12 dimensions of safety culture, and differences in response rate or scores were identified compared to the mean of the dimensions. The heterogeneous of safety culture's situation among Southeast Asian countries, both in practice and in research, can be explained since patient safety policy and its application are not prioritized as much as they are in developed countries in the priority compared to the developed countries. However, Vietnam, Cambodia, Myanmar, and Laos are the priority countries for South Korea's official healthcare development assistance in the Southeast Asia region. Vietnam, for instance, is an economically transitioning country; therefore, consolidated patient safety improvement by inducing patient safety culture in the provincial and central health system as well as strengthening project formulation to contribute to health policy formation are needed for sustainable development of the partner countries' health systems. It is recommended that more evidence-based proactive project planning and implementation be conducted to integrate patient safety culture into the health systems of developing countries, toward health policy on patient safety and quality service for the attainment of sustainable development goals in South Korea's development cooperation.

## Introduction

Patient safety is a global concern at all levels of healthcare systems, and its main purpose is to reduce patient risks when providing healthcare services (1). Since adopting the resolution at the 55th World Health Assembly in 2002, the World Health Organization (WHO) has recommended its member states to make systematic amendments to improve patient safety culture and healthcare quality (2). The importance of patient safety, however, was emphasized in a 1999 report titled, “To err is human” (3). In 2006, the WHO Patient Safety committee reached a consensus to effectuate a global agenda for promoting patient safety research in developing, transitioning, and developed countries (1). Further, the World Alliance for Patient Safety, established in 2014, considered patient safety as one of the global common tasks and identified main action areas related to it (4). Patient safety is a priority issue for healthcare systems in both developed and developing countries.

The recommended building blocks of health systems are outlined as healthcare providers, essential medical equipment and medicines, service delivery systems, health finance, and governance. To accomplish the improved health and efficiency of a health system, health policy on patient safety, service quality, and access and coverage are indispensable (5). Particularly, patient safety culture is a factor affecting the health service quality of a healthcare institution (6, 7). In 2010, the South Korean society proactively demanded the enactment of the Patient Safety Act (PSA), which was finally enacted on January 28, 2015 (PSA, Act No. 13113) (8). This Act has been in force since July 29, 2016. As an important action plan in health policy, the improvement of awareness on patient safety and voluntary reporting of adverse events is implemented. Meanwhile, the health authority is focusing on strengthening the health care system through the establishment of a patient safety culture by accumulating reports on major incidents and providing them as reporting-and-learning opportunities to prevent safety accidents. Since joining the OECD-DAC in 2010, South Korea has strengthened health systems of underdeveloped countries in Southeast Asian countries through the health sector's official development assistance. However, there have been few studies that discuss the patient safety issues as health policy to strengthening health system in Southeast Asian countries.

Therefore, a systematic review of patient safety and quality in Southeast Asian countries recommended that comprehensive research on healthcare safety and quality are needed, and that patient safety interventions implemented in developed countries must also be directly applied in developing countries (9). In contrast, patient safety culture was recognized as a key factor to improve patient safety (10) and quality of care in healthcare organizations, and the creation of safety culture was the first approach to guide healthcare providers into patient safety. Patient safety is explained as “the product of individual and group values, attitudes, perceptions, competencies, and patterns of behavior that determine the commitment to, and the style and proficiency of, and organization's health and safety management;” however, the measurement of patient safety has varied in previous research and several recommendations have been made to develop a standard measurement tool on patient safety (11).

Over the last 10 years, patient safety culture has been one of the most critical factors for studies assessing patient safety and quality of healthcare services (9, 11–22). Although some research has compared Japan, Taiwan, and the United States (23), as well as East-Asian countries (24), scant research has addressed patient safety culture in Southeast Asia countries. These discrepancies in patient safety culture studies across countries could be explained by complex factors, such as socioeconomic factors, cultural contexts, educational readiness, health manpower training, and institutional support. Understanding the least-developed countries among this region is the priority concern in the official development assistance in the health sector of the Republic of Korea.

The Korea International Cooperation Agency and Korea Foundation for International Health Care have been supporting developing countries' health sector since 1990. The specific areas within these health sectors include maternal and child health, school health, control of infectious diseases, and strengthening of health systems. In the last 10 years, most of the top 10 recipient countries have been Southeast Asian countries, owing to their geographical proximity, cultural similarities, and other political considerations (25, 26). Therefore, it is necessary to consider patient safety issues in all healthcare settings in the partner Southeast Asian countries while also planning and implementing healthcare development cooperation projects for the protection of patients' rights and service improvement in developing countries. Research focusing on the similarities and differences in patient safety culture across Southeast Asian countries is necessary. This would allow them to conduct health sector projects with partner countries while considering their experiences and advancements in patient safety.

The purpose of this systematic review was to identify the current status of patient safety culture in Southeast Asian countries, which will provide evidence to develop international cooperative projects aimed at promoting patient safety culture for strengthening the health system through health policy development.

## Methods

### Study Design

This systematic review included studies on patient safety culture conducted in Southeast Asian countries published between January 2009 and March 2020.

### Systematic Review Protocol

The Preferred Reporting Items for Systematic Reviews and Meta Analyses (PRISMA) checklist was applied to enhance the reporting quality of the included reviews (27).

### Search Strategy

Medline/PubMed, Cumulative Index to Nursing and Allied Health Literature, and Embase databases were searched for potential articles. Furthermore, the WHO Institutional Repository for Information Sharing was included to retrieve any articles on patient safety issues in regional countries. A Thai database was added to include relevant studies published in domestic journals in English abstract, as well as any unpublished master's theses and doctoral dissertations identified through manual searches. Keywords for the search were followed by considering the populations, interventions, comparators, outcomes, and study designs (PICOS) in the search. For populations, we searched keywords as “Asia,” “Southeast,” (MeSH) “Southeast Asia,” (as well as the names of all countries in Southeast Asia), and interventions as “safety management,” (MeSH) “safety culture(s),” “hazard management,” “hazard surveillance program(s),” “hazard control(s),” “patient safety” (MeSH), “safety climate,” and “safety communication.” However, we did not apply the every steps of PICOS after receiving guidance from a senior medical librarian who has experience consulting on systematic review studies at the Medical Library of Seoul National University College of Medicine in South Korea because our study could be more broadly searched by using the populations and interventions of PICOS.

### Inclusion and Exclusion Criteria

**Figure 1:**
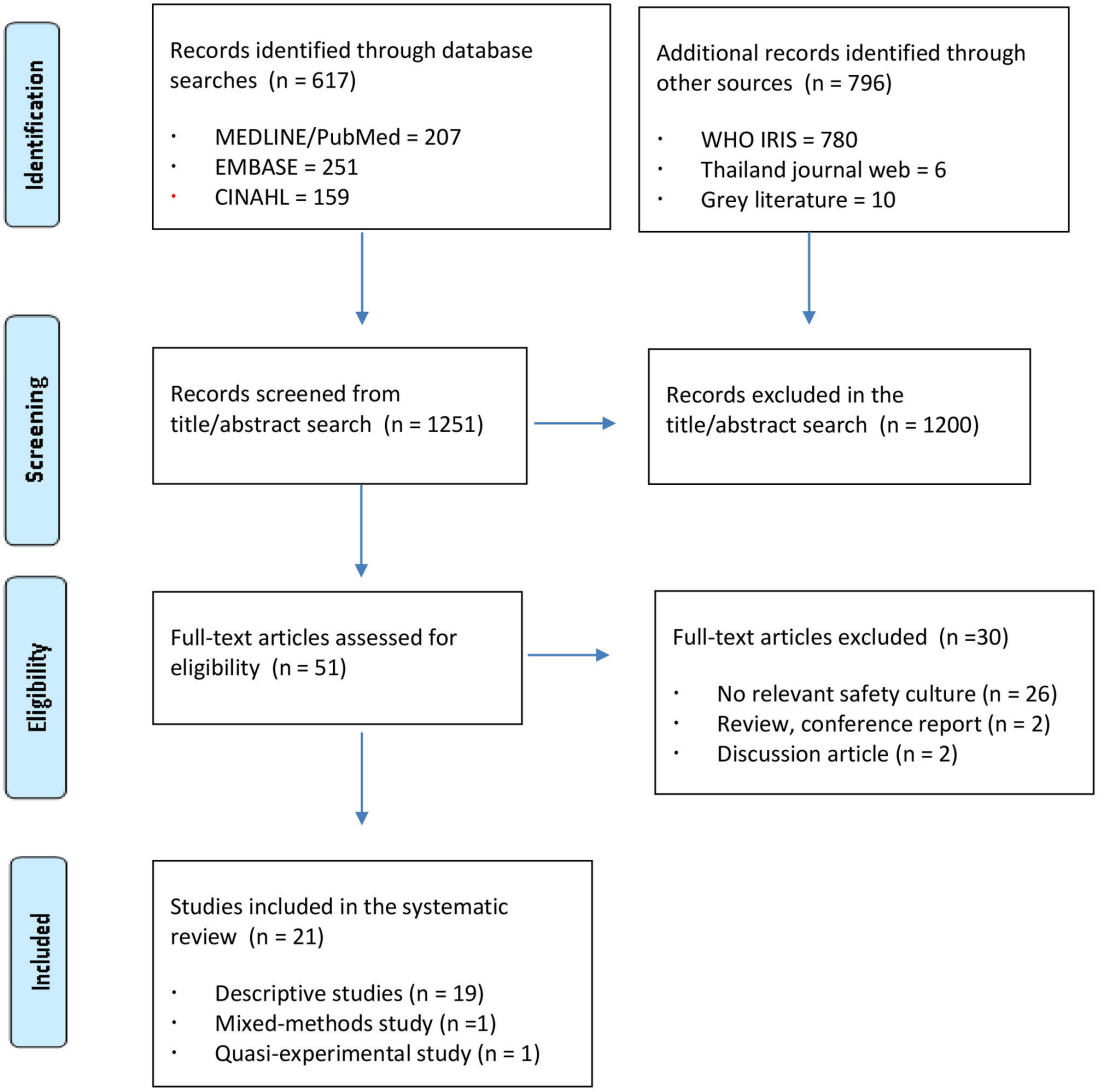
Flow diagram of the study selection process.

Articles were included if they (a) described patient safety culture, (b) were conducted in Southeast Asian countries, (c) were written in English, and (d) were published between January 1999 and March 2020. A total of 1,413 articles were identified. Furthermore, a domestic online database in Thailand was accessed for searching related studies; then, four studies written in Thai were converted in English. After removing duplicate articles, title and abstract screening was performed for 1,251 articles; of these, 1,200 articles that did not meet the inclusion criteria were excluded. Figure 1 illustrates the study selection process based on the PRISMA guidelines.

### Quality Assessment of Extracted Data

The Joanna Briggs Institute (JBI) tool, comprising eight items to determine the validity of descriptive cross-sectional studies, was used (28). Nine items for quasi-experimental study and mixed-methods appraisal tool of 13 categories were applied for each study (29). Two reviewers (SK and HT) independently assessed the extracted data. Disagreement was resolved through discussions between the reviewers. Excluded studies did not examine patient safety culture as the main variable, or they only addressed it in the discussion as something for future consideration. Finally, 21 articles included in this review.

### Analysis Strategy

The extracted data were analyzed by research design, number of participants, tools, research findings, and safety culture as main variables. General characteristics of the included studies were reviewed by publication year, research field, level of research, theoretical framework, and study setting using frequencies and percentages. Measurement tools were classified by names of tools and developers, subcategories, and item numbers of tools using frequencies and percentages; their reliability coefficients were reported. The findings were then sorted by research design. All the extracted studies were descriptive research except two that employed quasi-experimental research designs. The descriptive studies were classified into cross-sectional and mixed-methods design.

Suggestions for future partnership projects, recommendations, and the status of partner countries in Southeast Asia regarding South Korea's official development assistance were also comprehensively evaluated.

## Results

### Characteristics of Reviewed Studies

#### Study Country and Study Design

The 21 studies were evaluated and selected for analysis in process of the quality assessment, which are summarized in Tables 1A,B. Based on the JBI tool, seven of nine questions were answered “yes” in an included review with two quasi-experimental studies for assessing validity because of the lack of control group and having one measurement for evaluation effect of intervention (31, 34). In addition, in one review that employed mixed methods (13), 11 of 12 questions were answered “yes” because of unclear regarding to appropriate consideration given to how findings relate to researchers' influence.

**Table 1A:** Summary of critical evaluation of reviewed studies of cross-sectional design with questions (*n* = 19).

**Evaluation questions**	**Number of articles**			
	**Yes**	**No**	**U**	**N/A**
1. Were the criteria for inclusion in the sample clearly defined?	19	0	0	0
2. Were the study participants and the setting described in detail?	18	0	1	0
3. Was the exposure measured in a valid and reliable way?	17	0	3	0
4. Were objective, standard criteria used for condition measurement?	13	4	2	0
5. Were confounding factors identified?	16	3	0	0
6. Were strategies to deal with confounding factors stated?	7	12	0	0
7. Were the outcomes measured in a valid and reliable way?	2	16	0	1
8. Was appropriate statistical analysis used?	19	0	0	0

*U, unclear; N/A, not available*.

**Table 1B:** Results of the critical evaluation of each study.

**References**	**Q1**	**Q2**	**Q3**	**Q4**	**Q5**	**Q6**	**Q7**	**Q8**	**Q9**	**Q10**	**Q11**	**Q12**
Aini (30)	Y	Y	Y	Y	Y	Y	N/A	Y	–	–	–	–
Wijaya et al. (31)^྿྿^	Y	Y	Y	N	N	Y	Y	Y	Y	–	–	–
Setiowati (21)	Y	Y	U	U	Y	N	Y	Y	–	–	–	–
Buhari et al. (22)	Y	Y	Y	Y	Y	N	N	Y	–	–	–	–
Harsul et al. (32)	Y	Y	Y	Y	Y	N	N	Y	–	–	–	–
Iriviranty (14)^྿^	Y	Y	Y	Y	Y	N	Y	N	Y	Y	Y	Y
Kusumawati et al. (33)	Y	U	Y	Y	Y	Y	N	Y	–	–	–	–
Wijaya et al. (34)^྿྿^	Y	Y	Y	N	N	Y	N	Y	Y	–	–	–
Samsuri et al. (35)	Y	Y	Y	N	Y	Y	N	Y	–	–	–	–
Alex Kim et al. (20)	Y	Y	Y	N	N	N	N	Y	–	–	–	–
Odu et al. (36)	Y	Y	Y	Y	Y	Y	N	Y	–	–	–	–
Jabonete and Concepcion (17)	Y	Y	Y	Y	Y	N	N	Y	–	–	–	–
Ramos and Calidgid (19)	Y	Y	Y	Y	Y	N	N	Y	–	–	–	–
Koh et al. (37)	Y	Y	U	N	N	N	N	Y	–	–	–	–
Phasinee Koetbungphra (38)	Y	Y	Y	Y	Y	Y	N	Y	–	–	–	–
Potaya (39)	Y	Y	Y	N	N	N	N	Y	–	–	–	–
Sukhnim et al. (40)	Y	Y	Y	Y	Y	N	N	Y	–	–	–	–
Sayamol (41)	Y	Y	Y	U	Y	N	N	Y	–	–	–	–
Somporn (42)	Y	Y	Y	Y	Y	N	N	Y	–	–	–	–
Luong (16)	Y	Y	Y	Y	Y	Y	N	Y	–		–	–
Nguyen (15)	Y	Y	Y	Y	Y	N	Y	Y	–	–	–	–

྿, *mixed method design*;

྿྿, *quasi-experimental design; Y, yes; N, no*.

Meeting inclusion criteria of 21 reviewed studies were summarized in Tables 2A–C. Of the 21 studies reviewed, the studies employed cross-sectional (*n* = 18), quasi-experimental study (*n* = 2), and mixed-methods (*n* = 1) designs. Studies were conducted in six Southeast Asian countries: Indonesia (*n* = 8), Thailand (*n* = 5), Malaysia (*n* = 3), Singapore (*n* = 1), Vietnam (*n* = 2), and the Philippines (*n* = 2). The reviewed studies were published between 2013 and 2020, and cross-sectional research design was applied to all reviewed ones as well as one mixed-methods design to examine the status of patient safety culture in Southeast Asia (Table 2A).

**Table 2A:** Characteristics of patient safety culture and major findings (*N* = 21).

**References**	**Country**	**Study design**	**Setting**	**Participant**	**Instrument**	**Major findings**		
						**Positive results**	**Negative results**	**Predictors of safety culture**
Aini (30)	Indonesia	Cross-sectional	Individual hospital	Nurses (*N* = 149)	The Modify Safety Attitudes Questionnaire	Not applicable	Not applicable	The significance values of workload and work stress were both significant (*p* < 0.001)
Wijaya et al. (31)	Indonesia	Quasi-experimental study	Individual hospital	Healthcare providers (Intervention group = 87 Control group = 103)	The Hospital Survey on Patient Safety Culture	Teamwork within unit (80%) Supervisor/manager expectations and actions promoting patient safety (77%) Organizational learning and continuous improvement (75%)	Handoffs and transitions (63%) Non-punitive responses to an error (63%) Staffing (68%)	The treatment significantly increased the patient safety culture (β = 0.738, SE = 0.258, *p* = 0.007)
Setiowati (21)	Indonesia	Cross-sectional	Individual hospital	Head nurses (*N* = 30)	Patient Safety Culture Questionnaires developed by the researcher	Most participants were enough to apply patients' safety culture (64.5%)	Head nurses had low level of patients' safety culture application (7.6%)	There was a relationship between head nurses' transformational leadership and the implementation of patient safety culture
Buhari et al. (22)	Indonesia	Cross-sectional	Two accredited hospitals	Nurses (*N* = 226)	Safety Attitudes Questionnaire	Teamwork climate (73%) Job satisfaction (68.1%)	Working condition (44.2%) Stress recognition (52.2%)	Significant relationships were found between teamwork, safety culture, stress, management, and working condition with the implementation of patient safety practices (*p* < 0.001 to 0.017), whereas job satisfaction was non-significantly related to patient safety (*p* = 0.928)
Harsul et al. (32)	Indonesia	Cross-sectional	Individual hospital	Nurses (*N* = 100)	The Hospital Survey on Patient Safety Culture	Feedback and communication about error (57%)	Overall perceptions of safety (30%) Frequency of event reporting (48%)	Self-efficacy was non-significantly correlated with the culture of patient safety incident reporting (*p* = 0.116)
Iriviranty (14)	Indonesia	Mixed-methods		Healthcare providers (*N* = 152)	The Hospital Survey on Patient Safety Culture	Teamwork within units (91.67%) Organizational learning and continuous improvement (89.8%) Supervisor/manager expectations and actions promoting patient safety (73.03%)	Staffing (22.7%) Non-punitive responses to an error (37.13%) Hospital handoffs and transitions (52.98%)	Not applicable
Kusumawati et al. (33)	Indonesia	Cross-sectional	Three hospitals	Nurses (*N* = 400)	The Hospital Survey on Patient Safety Culture	Teamwork within units (82.84%) Organizational learning and continuous improvement (79%) Feedback and communication about error (76.4%)	Staffing (64.5%) Overall perceptions of patient safety (70.42%) Hospital handoffs and transitions (73.5%)	Significant relationships existed between patient safety culture and nurses' attitudes toward incident reporting (r = 0.838, *p* = 0.005)
Wijaya et al. (34)	Indonesia	Quasi-experimental study	Three hospitals	Healthcare providers (*N* = 484)	The Hospital Survey on Patient Safety Culture	Teamwork within units Organization learning-continuous improvement	Teamwork across units Handoffs and transitions	Shift schedule realignment was associated with patient cultural safety
Samsuri et al. (35)	Malaysia	Cross-sectional	3 public hospitals and 27 health clinics	Pharmacists (*N* = 117)	Safety Attitudes Questionnaire	Stress recognition (58.1%) Job satisfaction (46.2%)	Working conditions (15.4%) Safety climate (33.3%)	Not applicable
Alex Kim et al. (20)	Malaysia	Cross-sectional	Individual hospital	Healthcare providers (*N* = 500)	The Hospital Survey on Patient Safety Culture	Organizational learning and continuous improvement (80%)	Non-punitive responses to an error (18%) Staffing (18%)	Not applicable
Odu et al. (36)	Malaysia	Cross-sectional	Individual university	Educators (*N* = 44)	The Modify Safety Attitude Questionnaire	42.5% had positive attitudes toward safety culture	27.5% participants had good knowledge of safety culture, and 32.5% practiced safety culture	Factors that were significantly associated with safety culture practice were job title (*p* =0.041) and length of service (*p* = 0.010). Age (*p* = 0.039) was significantly associated with safety practice
Jabonete and Concepcion (17)	Philippines	Cross-sectional	4 hospitals	Healthcare providers (*N* = 530)	Manchester Patient Safety Culture Assessment Tool	At proactive level, personnel management (69%), system errors and individual responsibility (66%), and learning and effecting change (61%)	At proactive level, dimensions of patient safety culture was low level including priority given to safety (47%), recording incidents (44%), and teamwork (40%)	Age group was significantly different among healthcare providers who perceived safety culture at reactive (F-5.45), bureaucratic (F-4.26), and proactive (F-3.66) maturity levels. Job position was found significantly different to those who perceived it at generative (F-3.95) level. Only participants who have perceived safety culture at reactive (F-2.26) level have significant differences in their scores together with length of experience at reactive (F-2.86) level. A significant difference was found to type of hospital to almost all safety culture level except at bureaucratic level.
Ramos and Calidgid (19)	Philippines	Cross-sectional	Individual hospital	Nurses (*N* = 292)	The Hospital Survey on Patient Safety Culture	Teamwork within units (91.50%) Organizational learning and continuous improvement (86.89%) Supervisor/manager expectations and actions promoting patient safety (67.34%)	Non-punitive responses to an error (17.65%) Staffing (27.55%) Overall perceptions of safety (50.78%)	Not applicable
Koh et al. (37)	Singapore	Cross-sectional		Healthcare providers (*N* = 600)	Patient Safety Culture Questionnaires developed by the researcher	88.0 and 85.6% agreed that clinical quality and patient safety are important and relevant to their work	36.2% of participants intervened when they see unsafe practice and 27.2% saw the importance of reporting near-miss events	Not applicable
Phasinee Koetbungphra (38)^*^	Thailand	Cross-sectional	Individual hospital	Healthcare providers (*N* = 176)	The Hospital Survey on Patient Safety Culture	Supervisor/manager expectations and actions promoting patient safety (M = 3.91 ± 0.51) Organizational learning and continuous improvement (M = 3.82 ± 0.46) Teamwork within units (M = 3.80 ± 0.50)	Staffing (M = 3.43 ± 0.59) Hospital management support for patient safety (M = 3.63 ± 0.62) Non-punitive response to an error (M = 3.54 ± 0.63)	Administrators, teamwork, employees' responsibilities of patient safety, work environment, and experience of receiving training on patient safety predicted patient safety culture
Potaya (39)^*^	Thailand	Cross-sectional	Individual hospital	Healthcare providers (*N* = 664)	The Hospital Survey on Patient Safety Culture	Teamwork within units (M = 4.27 ± 0.48) Organizational learning and continuous improvement (M = 4.11 ± 0.42) Supervisor/manager expectations and actions promoting patient safety (M = 4.09 ± 0.51)	Non-punitive responses to an error (M = 3.06 ± 0.85) Teamwork across hospital units (M = 3.07 ± 0.69) Staffing (M = 3.23 ± 0.70)	Executive nurses had significantly higher patient safety culture scores than did staff nurses (*p* = 0.006)
Sukhnim et al. (40)	Thailand	Cross-sectional	Individual hospital	Healthcare providers (*N* = 380)	The Hospital Survey on Patient Safety Culture	Organizational learning and continuous improvement	Staffing (M = 3.10 ± 0.74) Non-punitive response to an error (M = 3.13 ± 0.90) Hospital handoffs and transitions (M = 3.12 ± 0.75)	Not applicable
Sayamol (41)^*^	Thailand	Cross-sectional	Individual hospital	Nurses (*N* = 102)	Patient Safety Culture Questionnaires was modified by the researcher based on framework of Nieva and Sorra (43)	Hospital handoffs and transitions (M = 4.48 ± 0.57) Communication openness (4.47 ± 0.46) Feedback and communication about errors (M = 4.22 ± 0.69)	Non-punitive responses to an error (M = 3.15 ± 0.92) Staffing (M = 2.08 ± 0.82) Hospital management support for patient safety (M = 3.19 ± 0.43)	A significant positive and moderate correlation was found between patient safety culture and nursing service quality (*r* = 0.462, *p* < 0.01).
Somporn et al. (42)^*^	Thailand	Cross-sectional	Regional and general hospitals	Nurses (*N* = 500)	The Hospital Survey on Patient Safety Culture	Supervisor/manager expectations and actions promoting patient safety Teamwork within units	Supervisor/manager expectations and actions Teamwork across units	Teamwork in support for safety culture did non-significantly differ (*p* = 0.11) between nurses from regional hospitals and those from general hospitals
Luong (16)	Vietnam	Cross-sectional	10 hospitals	Healthcare providers (*N* = 1,500)	The Hospital Survey on Patient Safety Culture	Teamwork within hospital units (81%) Organizational learning and continuous improvement (75%) Hospital management support for patient safety (72%)	Non-punitive response to an error (44%) Hospital handoffs and transitions (47%) Staffing (55%)	Not applicable
Nguyen (15)	Vietnam	Cross-sectional	Two urban public hospitals	Nurses (*N* = 189)	The Safety Attitudes Questionnaire	Teamwork climate (51.4–93.8) Working conditions (50.0–94.1)	Stress recognition (7.1–57.1) Safety climate (21.1–81.3)	Not applicable

* *Originally written in Thai language with English abstract and converted to English for analysis*.

#### The Measurement of Aspects on Patient Safety Culture

Study settings on patient safety culture included hospital and community clinics. At the hospital level, the number of hospitals surveyed ranged from 1 to 10 hospitals. One study focused on both hospital and clinical settings (35).

Thirteen studies measured the concept of safety culture using the hospital survey on patient safety culture [HSOPSC; (14, 16, 19, 20, 31–34, 38–42)]; three used the Safety Attitude Questionnaire [SAQ; (15, 22, 35)]; two used the modified SAQ (30, 36); two developed their questionnaire on patient safety culture (21, 37); and one used the Manchester Patient Safety Culture Assessment Tool [MaPSCAT; (17)]. The tools on patient safety culture using the HSOPSC were categorized into sub-concepts of safety culture in 12 areas: supervisor expectations and actions promoting safety, organizational learning improvement, teamwork within hospital units, communication openness, feedback and communication about errors, non-punitive response to errors, staffing, hospital management support, teamwork across hospital units, hospital handoffs and transitions, frequency of event reporting, and overall perceptions of safety. SAQ domains included teamwork climate, safety climate, job satisfaction, stress recognition, perceptions of management, and working condition.

Reviewed studies provided questionnaires to participants including healthcare providers and educators. Ten studies focused on healthcare providers' perception on safety culture in hospital settings: nine studies included those of head nurses and staff nurses (15, 19, 21, 22, 30, 32, 33, 41, 42), one study included pharmacists (35), and one study included educators at a medical university (36). Two studies evaluated interventions for promoting patient safety culture among healthcare providers (31, 34).

### Major Findings Concerning Patient Safety Culture

Different conceptual frameworks and instruments were utilized for assessing the level of patient safety culture; however, in most studies, most of the positive rated scores focused on six dimensions of patient safety culture following the HSOPSC tool. The scores were calculated by percent-positive scores which combined percentage of respondents who answered “strongly agree,” or “agree,” or “always,” or “most of the time,” following the Agency for Healthcare Research and Quality guidelines. Only studies from Thailand applied mean scores within five points (see Table 2B). Among the extracted articles, the lists of four to six dimensions of patient safety culture with low or high scores were selected and included in the data analysis. Higher scores of patient safety culture dimensions applied to HSOPSC were considered as positive patient safety culture dimensions while lower score dimensions were considered as negative patient safety culture dimensions. Seven dimensions were classified using HSOPSC studies as positive results within patient safety culture: supervisor and/or manager expectations and actions promoting patient safety (14, 19, 31, 38–40, 42), organizational learning and continuous improvement (14, 16, 19, 20, 31, 33, 34, 38–40), teamwork within units (14, 16, 19, 31, 33, 34, 38, 39, 42), feedback and communication about errors (24, 32, 40, 41), communication openness (41), handoffs and transitions (41), and hospital management support for patient safety (16). In the articles that used the SAQ, dimensions of positive results included teamwork climate (22), job satisfaction (22, 30, 35), stress recognition (35), teamwork climate (15), and working conditions (15). However, one study based on the cumulative calculation of whole wards showed that job satisfaction was the component with the highest percentage, implying that job satisfaction was the most important factor related to patient safety attitude (30). One study applied the MaPSCAT and found that positive outcomes comprised personnel management, system errors, individual responsibility, and learning and effecting change at the proactive level. Odu et al. (36) showed that 42.5% of educators recognized having positive attitudes toward safety culture. Additionally, one study indicated that 88.0 and 85.6% of healthcare providers agreed that clinical quality and patient safety were important and relevant to their work, respectively [(37); see Table 2C].

**Table 2B:** The percent-positive scores of dimensions of patient safety culture as measured by the Hospital Survey on Patient Safety Culture (*N* = 13).

**References**	**Country**	**Supervisor/manager expectations and actions promoting patient safety**	**Organizational learning and continuous improvement**	**Teamwork within unit**	**Communication openness**	**Feedback and communication about error**	**Non-punitive response to an error**	**Staffing**	**Hospital management support for patient safety**	**Teamwork across hospital units**	**Hospital handoffs and transitions**	**Overall perceptions of safety**	**Frequency of event reporting**
Wijaya et al. (31)	Indonesia	77%	75%	80%	73%	72%	63%	68%	72%	67%	63%	72%	71%
Harsul et al. (32)	Indonesia	N/A^*^^*^	N/A^*^^*^	N/A^*^^*^	N/A^*^^*^	57%	N/A^*^^*^	N/A^*^^*^	N/A^*^^*^	N/A^*^^*^	N/A^*^^*^	30%	48%
Iriviranty (14)	Indonesia	73.03%	89.8%	91.67%	68.6%	72.07%	37.13%	22.7%	84.77%	69.76%	52.98%	67.35%	70%
Kusumawati et al. (33)	Indonesia	74.44%	79%	82.84%	73%	76.4%	72.79%	64.5%	76.6%	75.2%	73.5%	70.42%	73.69%
Wijaya et al. (34)	Indonesia	N/A^*^^*^	N/A^*^^*^	N/A^*^^*^	N/A^*^^*^	N/A^*^^*^	N/A^*^^*^	N/A^*^^*^	N/A^*^^*^	N/A^*^^*^	N/A^*^^*^	N/A^*^^*^	N/A^*^^*^
Alex Kim et al. (20)	Malaysia	N/A^*^^*^	80%	N/A^*^^*^	N/A^*^^*^	N/A^*^^*^	18%	23%	N/A^*^^*^	N/A^*^^*^	N/A^*^^*^	50.1%	N/A^*^^*^
Ramos and Calidgid (19)	Philippines	67.34%	86.89%	91.50%	48.36%	76.32%	17.65%	27.55%	60.28%	68.77%	55.97%	50.78%	54.12%
Koh et al. (37)	Singapore	88.0% and 85.6% agreed that CQPS was important and relevant to their work, respectively. Only 36.2% will intervene when they see unsafe practice and 27.2% see the importance of reporting near-miss events											
Phasinee Koetbungphra (38)^*^	Thailand	3.91 ± 0.51	3.82 ± 0.46	3.80 ± 0.50	3.60 ± 0.67	3.76 ± 0.65	3.54 ± 0.63	3.430.59 ±	3.63 ± 0.62	3.70 ± 0.50	3.71 ± 0.44	3.70 ± 0.38	3.79 ± 0.79
Potaya (39)^*^	Thailand	4.09 ± 0.51	4.11 ± 0.42	4.27 ± 0.48	3.83 ± 0.52	3.69 ± 0.53	3.97 ± 0.77	3.23 ± 0.70	3.90 ± 0.48	3.07 ± 0.69	3.06 ± 0.85	3.74 ± 0.32	3.78 ± 0.48
Sukhnim et al. (40)^*^	Thailand	3.85 ± 0.67	3.89 ± 0.60	3.76 ± 0.69	3.60 ± 0.67	3.83 ± 0.60	3.13 ± 0.90	3.10 ± 0.74	3.40 ± 0.65	3.68 ± 0.61	3.12 ± 0.75	3.47 ± 0.56	3.27 ± 0.97
Sayamol (41)^*^	Thailand	3.63 ± 0.72	3.73 ± 0.83	3.91 ± 0.73	4.47 ± 0.46	4.22 ± 0.69	3.15 ± 0.92	2.08 ± 0.82	3.19 ± 0.43	3.61 ± 0.69	4.48 ± 0.57	3.68 ± 0.42	4.00 ± 0.80
Somporn et al. (42)	Thailand	The average perception scores of patient safety culture regarding management of safety, working safety, and communication within units were at a high level and working of the supervisor/head of unit was at a moderate level											
Luong (16)	Vietnam	69%	75%	81%	62%	69%	44%	55%	72%	61%	47%	66%	66%

* *The scores of these studies described as means because authors calculated means rather than percent-positive scores. N/A, not applicable*.

**Table 2C:** Range of the dimensions of patient safety culture as measured by the Safety Attitudes Questionnaire (*N* = 5).

**References**	**Country**	**Teamwork climate**	**Safety climate**	**Job satisfaction**	**Stress recognition**	**Perceptions of management**	**Working conditions**	**Whole scale**
Aini (30)	Indonesia	N/A^*^	N/A^*^	highest	N/A^*^	N/A^*^	N/A^*^	N/A^*^
Buhari et al. (22)	Indonesia	73.0%	55.3%	68.1%	52.2%	59.7%	44.2%	N/A^*^
Samsuri et al. (35)	Malaysia	38.5%	33.3%	46.2%	58.1%	29.9%	15.4%	20.5%
Odu et al. (36)	Malaysia		27.5% of the respondents had good knowledge on safety culture, about 42.5% of the respondents had positive attitudes toward safety culture. The practice of safety culture was reported by only 32.5% of the respondents with less than half displaying good practice toward safety culture					
Nguyen (15)	Vietnam	51.4–93.8%	21.1–81.3%	42.9–100.0%	7.1–57.1%	21.4–100.0%	50.0–94.1%	N/A^*^

* *The article did not include the result of each dimension. N/A, not applicable*.

Negative dimensions were divided by analyzing the lowest dimension of HSOPSC studies and identified as handoffs and transitions (14, 16, 31, 33, 34, 40), non-punitive responses to an error (14, 16, 19, 20, 31, 38–41), staffing (14, 16, 19, 20, 31, 33, 38–41), supervisor/manager expectations and actions (42), teamwork across units (34, 39, 42), overall perceptions of safety (19, 32, 33), hospital management support for patient safety (38), and frequency of event reporting (32). In the dimension of safety culture using the SAQ, the negative results involved working conditions (22, 35), stress recognition (15, 22), and safety climate (15, 35). One study mentioned that 27.5% of participants had a good knowledge of safety culture, and 32.5% practiced safety culture (36). Another study (17) indicated that, at a proactive level, the following prevalence: priority given to safety (47%), recording incidents (44%), and teamwork (40%). In addition, one recognized that 36.2% of participants intervened when they saw unsafe practice and 27.2% saw the importance of reporting near-miss events (37).

Twelve out of 21 studies determined factors influencing patient safety culture; consequently, five dimensions were classified as predictors: organization and management, work environment, care delivery, team factors, and individual factors. Organization and management factors included perceptions of management (22), administrators (38), and nursing service quality (41). Work environment was reported as a main factor influencing patient safety culture (22, 30, 34, 36, 38). Team factors (22, 38) and care delivery (31) were proposed as predictors of patient safety outcomes. Individual factors included age group (17, 36), job position (17, 21, 36, 39), implementation of patient safety practices (22), attitudes toward incident reporting (33), attitude and responsibility toward patient safety (36, 38), and experience of receiving training on patient safety (38).

## Discussion

The status of patient cultural safety in Southeast Asian countries was highlighted in this review. Most studies used either the HSOPSC or the SAQ to measure safety culture, which mirrored previous findings (11). Ten Vietnamese hospitals used the HSOPSC, and the average percentage outcome for safety culture was positive (58.9%), which was less than that reported among American hospitals (16). A study of patient safety culture in hospitals in the Philippines using the MaPSCAT revealed that recording and reviewing safety accidents was essential for the formation of a positive organizational culture (17).

Based on the dimensions of patient safety culture in Southeast Asian countries, the importance of patient safety culture has been recognized in healthcare systems. However, this review showed that patient cultural safety was mentioned in five Southeast Asian countries: Laos, Cambodia, Myanmar, Brunei, and East Timor. Therefore, researchers should consider assessing patient safety culture in those countries or provide interventional programs for healthcare providers to enhance health and safety awareness among those countries. Additionally, most studies utilized a descriptive quantitative design to identify the status of patient safety culture, and only one study focused on intervention effectiveness; thus, interventional programs related to this issue need to expand into the health system in Southeast Asian countries.

In this review, supervisor and/or manager expectations and actions promoted patient safety, organizational learning and continuous improvement, teamwork within units, and teamwork climate, and working conditions are a positive dimensions of patient safety culture. It was evident that healthcare providers in Southeast Asian countries are aware of the need to have supportive health organizations, team collaboration, and continue educational training. Furthermore, safety behaviors regarding stress recognition and feedback and communication about errors were concentrated to improve workplace health and safety. These findings are similar to previous studies in developed and developing countries.

Teamwork within units, organizational learning, and continuous improvement have been identified as crucial dimensions of patient safety culture (44, 45). In a study conducted in Peru, the support given by administration for patient safety, non-punitive report of errors, and frequency of reported incidents were dimensions of patient safety with low percentage of healthcare providers' positive responses (46). Raeissi et al. (47), who examined an Iranian hospital, found that organizational learning continuous improvement, teamwork within hospital units, and support from hospital management for patient safety were positive factors for patient safety implementation. In Taiwan, working conditions and stress recognition positively affected patient safety (48). Ricklin et al. found that teamwork within units and supervisor/manager expectations and actions promoting patient safety were positively rated by healthcare providers in Switzerland (49).

Regarding the negative dimensions to patient safety culture in this review, handoffs and transitions, non-punitive responses to an error, patient safety reporting, and staffing were generally considered barriers to patient safety culture. It was evident that cultural safety activities regarding developing patient safety report systems should be promoted and cultural safety educational programs for healthcare professionals should be encouraged. Similar findings were reported by Reis et al. (44), who indicated that non-punitive responses to an error, staffing, handoffs, transitions, and teamwork across units were barriers to patient safety culture. Elmontsri et al. (45), who examined Arab countries, found that non-punitive responses to an error was the least practiced in healthcare organizations. In Peru, staffing and non-punitive responses to an error were barriers to safety culture (46). In an Iranian hospital, feedback and communication concerning errors, communication openness, staffing, and non-punitive responses to an error were also identified as barriers (47). Khate et al. (50), who examined Jordanian hospitals, showed that communication openness, staffing, handoffs and transitions, non-punitive responses to errors, and teamwork across units were areas that needed improvement. In Palestine, Elsous et al. revealed that working conditions and stress recognition required improvements to enhance the patient safety culture (51).

Moreover, the overall prevalence of patient safety culture ranged from low to moderate in this review. This suggests that patient safety culture should be further promoted among healthcare providers in Southeast Asian countries. These results are like those of studies conducted in developed and developing countries. In Peru, the degree of perceived patient safety was low among healthcare providers (46). Mayeng and Wolvaardt (52) indicated that medical doctors had negative perceptions of all the patient safety dimensions, while half of the healthcare providers in Hungarian hospitals indicated that their patient safety practices were acceptable (53). Moreover, frequencies of reported events, teamwork across units, and handoffs and transitions were all scored low in Switzerland (49). Additionally, a study conducted in a primary care setting in Yemen showed that the overall patient safety culture was low owing to lack of formal safety and quality management systems (54). Similarly, the WHO reported that lack of safety culture and attitudes were common problems in Southeast Asia (55).

This comparative analysis of 21 studies revealed that there were two factors affecting patient safety culture: systematic factors and human factors. The systematic factors included organization and management, work environment, care delivery, and team factors, while human factors included the main variables affecting safety culture. An Indonesian study reported the determinants of patient safety implementation among nurses as teamwork, safety culture, stress recognition and management, working conditions, and standard work guidelines (33). Kuosmanen et al. (56) concluded that implementation of a patient safety incident reporting system positively influenced patient safety culture. Moreover, Dirik and Intepeler (57) found that the work environment was related to patient safety culture and teamwork within units was an important factor of patient safety culture in Jordanian hospitals. Healthcare professionals' age, position, total years of experience, experience working in university hospitals, and working hours were the key elements of human factors affecting patient safety culture (50, 51). Perception of the work environment, attitudes toward incident reporting, and patient safety culture were all positively associated (58).

## Conclusions

This study explored the status of patient safety culture in Southeast Asian countries to identify differences in health policy. The level of safety culture was low to moderate in the context of system and human factor dimensions. There was also low volume of research among these countries, and most was published in Indonesia and Thailand. However, this review had some limitations, such as the exclusion of research written in non-English, which could have resulted in failing to include other relevant studies. Countries with fewer reported studies on patient safety culture reveal that their safety culture is negligible, and that regulation similar to the Patient Safety Act is a requirement of public safety for their citizens.

For the future consideration of future health system cooperation, WHO's recommendations for research priorities regarding patient safety, economic status, safety culture, and communication are appropriate as tracer topics on the progress of healthcare system development (1) because they are key priorities in both developed and developing countries for the strengthening of health systems. Patient safety issues in South Korea's development cooperation in the health sector have not been considered a priority project for the partner countries' sustainable health system policy formation. In this systematic review, the discrepancies between the importance of patient safety culture both in practice and in research were explained by the fact that most of the studies were conducted in developed countries. However, Vietnam, Cambodia, Myanmar, Laos, and Philippines are priority countries for South Korea's official healthcare development assistance in the Southeast Asia region, and Vietnam is an economically transitioning country. Therefore, consolidating patient safety improvement by inducing patient safety culture in the provincial and central health system, as well as strengthening health policy formulation are needed for sustainable development of the partner countries' health systems.

Thus, we recommend that more evidence-based proactive project planning and implementation be conducted to integrate patient safety culture into healthcare and for the attainment of sustainable development goals in South Korea's development cooperation. Although we examined the developed and some developing countries in Southeast Asia, increased policy formation regarding patient safety, raising awareness of patient safety for healthcare providers, patients and the community are needed as well. For South Korea's partner countries of official development cooperation, such as Vietnam, Lao PDR, Cambodia, and Philippines, without quality of service and patient safety, it is impossible to expect health systems to improve.

## Data Availability Statement

The raw data supporting the conclusions of this article will be made available by the authors, without undue reservation.

## Author Contributions

SK and N-JL conceived the study conception and design. SK and TH contributed to the data collection, data analysis, and manuscript draft. N-JL contributed to the overall quality of the draft manuscript and its revisions. All authors have read and approved the final manuscript.

## Conflict of Interest

The authors declare that the research was conducted in the absence of any commercial or financial relationships that could be construed as a potential conflict of interest.
